# Ultra-widefield color fundus photography in diabetic retinopathy: from panretinal assessment to multimodal integration

**DOI:** 10.3389/fmed.2026.1845156

**Published:** 2026-06-03

**Authors:** Ruolin Fang, Ruixuan Xu, Zhipeng Wu, Yuhua Tong

**Affiliations:** 1 Jinhua Graduate Joint Training Base, Zhejiang Chinese Medical University, Zhejiang, China; 2 Clinical Medical College, Hainan Medical University, Haikou, China; 3 Women and Children’s Health Care Hospital of Luohu, Shenzhen, Guangdong, China; 4 Department of Ophthalmology, Quzhou People’s Hospital, The Quzhou Affiliated Hospital of Wenzhou Medical University, Quzhou, Zhejiang, China

**Keywords:** artificial intelligence, color fundus photography, diabetic retinopathy, multimodal integration, panretinal assessment, ultra-widefield imaging

## Abstract

Diabetic retinopathy (DR) is the leading cause of vision loss among working-age adults. Early screening and precise staging are crucial for delaying disease progression. Although the traditional Early Treatment of Diabetic Retinopathy Study (ETDRS) 7-field method is the gold standard for grading, it covers only 35% of the retinal area, carrying a risk of missing peripheral lesions. Ultra-widefield color fundus photography (UWF-CFP) overcomes this limitation by capturing up to 200° of the retina in a single image, enabling comprehensive visualization of peripheral ischemia, neovascularization, and peripheral–posterior pole asymmetry lesions. In recent years, combining UWF-CFP with deep learning algorithms has achieved robust performance in automated DR screening, grading, and quantitative vascular analysis. When further integrated with ultra-widefield swept-source optical coherence tomography angiography (UWF SS-OCTA) or multimodal clinical data, these AI-driven methods can improve diagnostic consistency and staging accuracy. Moreover, because the retinal microvasculature mirrors systemic microcirculation, vascular parameters from UWF-CFP have shown correlations with diabetic nephropathy, coronary artery calcification, and stroke risk, highlighting a potential role in non-invasive assessment of systemic complication risks in diabetic patients. UWF-CFP is facilitating a shift in the DR assessment paradigm from ETDRS 7-field to full retinal assessment. Combined with artificial intelligence and multimodal data integration, UWF-CFP has the potential to contribute to a future care model that integrates ocular and systemic risk assessment, which we tentatively term “eye–system collaborative management,” though this paradigm remains to be validated in prospective studies.

## 1 Introduction

Diabetic retinopathy (DR), a major microvascular complications of diabetes, has become the leading cause of irreversible visual impairment among working-age populations worldwide (1, 2). The global prevalence of DR among people with diabetes is 22.27%, and it is projected that the number of affected individuals will increase to approximately 160.5 million by 2045 (3). Therefore, early detection and intervention remain crucial. The ETDRS standard 7-field fundus photography method has long been regarded as the gold standard for DR grading and research (4, 5). This method acquires 7 stereoscopic fundus photos with a 30° field of view, precisely covering the posterior pole to assess the location and extent of posterior pole lesions (6, 7). However, because it covers 35% of the total retinal area, peripheral lesions can be missed (8). Studies have shown that approximately 30%–40% of DR patients have more severe peripheral retinal lesions than posterior pole lesions, and this phenomenon is called “peripheral-posterior pole asymmetry” (9–11), defined as the presence of more severe DR lesions (e.g., microaneurysms, hemorrhages, or intraretinal microvascular abnormalities) in the peripheral retina (beyond the ETDRS 7-field area) than in the posterior pole, or the exclusive presence of lesions in the periphery without corresponding posterior pole involvement. Therefore, staging that relies exclusively on posterior pole images may systematically underestimate the true severity of DR.

**FIGURE 1:**
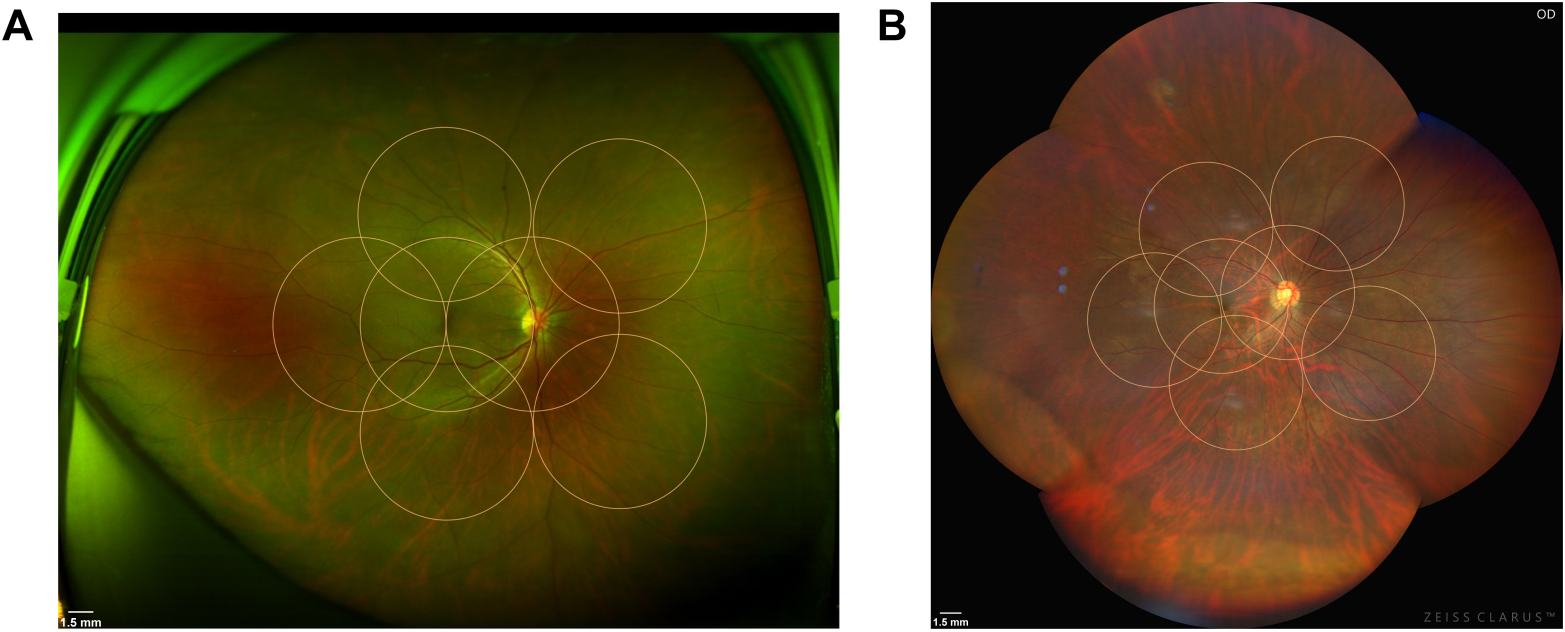
Comparison of two mainstream UWF-CFP systems with the coverage of ETDRS 7-field photography. **(A)** Representative UWF-CFP image from the Optos^®^ system, demonstrating the 200° pseudo-color view that covers approximately 82% of the retinal surface. **(B)** An analogous UWF-CFP image acquired with the Zeiss Clarus^®^ system (133° field of view) demonstrating true-color rendering capability. The seven circles in both images indicate the standard positions and extents of ETDRS 7-field photography (each field covers 30°, collectively covering approximately 35% of the retina). The seven fields are centered on: the macula, the optic disc, temporal to the macula, superior and inferior to the optic disc, and superior and inferior to the macula. This side-by-side comparison clearly and intuitively demonstrates the key technical trade-offs (field of view and color fidelity), and shows that over 60% of the retina surface falls outside the scope of traditional assessments. The scale bar in the lower left corner represents 1.5 mm.

The emergence of UWF-CFP now allows comprehensive assessment of retinal ischemia, neovascularization, and inflammatory changes. Currently, two main UWF-CFP devices are in clinical use: confocal scanning laser ophthalmoscopes (cSLO) represented by the Optos^®^ series and true color ultra-wide field photography technology represented by the Zeiss Clarus^®^ series. Figure 1 provides a side-by-side comparison of the retinal coverage and color rendering capabilities of these two systems, with the ETDRS 7-field area superimposed as a spatial reference. Quantitative specifications of both systems are detailed in Table 1. The Optos^®^ system uses two wavelengths of laser for scanning (12, 13). The green laser (532 nm) mainly images the inner retina and retinal pigment epithelium, while the red laser (633 nm) can penetrate the choroid to display deep structures. This dual-laser approach achieves a wide imaging range (up to 200°) and is largely unaffected by pupil size and mild media opacities (14, 15). In contrast, the Clarus^®^ system uses multi-spectral true color imaging with a high-resolution sensor (>5 million pixels), producing a 133° ultra-wide field of view with true color restoration (16). Its color representation is closer to the clinical slit-lamp view, facilitating accurate identification and stratified analysis of microaneurysms, hemorrhage points, and lipid exudation (17). Each technology has distinct advantages and limitations: Optos^®^ offers a wider field of view suitable for global peripheral assessment but generates pseudo-color images with lower color fidelity, which can affect qualitative evaluation of some lesions (18); Clarus^®^ provides superior color fidelity, excelling in detailed posterior pole observation, but its image quality depends more on pupil dilation and media clarity, and its single-field coverage is slightly narrower (19). UWF-CFP enables evaluation covering nearly 82% of the total retinal surface in one capture (20). Studies have confirmed its consistency with ETDRS seven-field photography in DR lesion assessment (21, 22).

**TABLE 1:** Comparison of key technical parameters between Optos^®^ and Zeiss Clarus^®^ ultra-widefield color fundus imaging systems.

Parameter	Optos^®^ (cSLO-based)	Zeiss Clarus^®^ (true-color)
Imaging principle	Confocal scanning laser ophthalmoscopy (cSLO)	Multispectral true-color non-mydriatic fundus camera
Field of view (single capture)	Up to 200° (approximately 82% of retinal surface)	133° (single capture)
Color mode	Pseudo-color (synthesized from green 532 nm and red 633 nm lasers)	True-color (multi-spectral, >5 million pixel sensor)
Resolution	Moderate; optimized for peripheral detection	High; detailed posterior pole visualization
Pupil requirement	Unaffected by small pupil size (≥2 mm)	Pupil dilation preferred; image quality more dependent on pupil size
Tolerance to media opacity	High; can penetrate mild cataracts or vitreous opacities	Lower; clarity of refractive media significantly affects image quality
Key strength	① Widest field of view ② Excellent for global peripheral assessment ③ Less affected by media opacity	① Superior color fidelity ② Closer to clinical slit-lamp appearance ③ Better for characterizing microaneurysms, hemorrhages, and exudates
Key limitation	① Pseudo-color may underestimate hard exudates, cotton-wool spots, and superficial hemorrhages ② Qualitative lesion assessment less accurate	① Narrower field than Optos^®^ ② More dependent on pupil dilation and clear media
Typical clinical application	Rapid screening for peripheral lesions (e.g., PPL, NVE) in diabetic retinopathy	Detailed posterior pole evaluation and lesion characterization; accurate grading of non-proliferative DR)

Although several recent reviews have summarized the application of UWF-CFP in DR, their frameworks largely remain confined to single-dimension summaries of imaging technology, lesion detection rates, or artificial intelligence (AI) model performance. These reviews often discuss imaging, AI, and systemic complications in isolation, lacking an integrated perspective. This review attempts to bridge these gaps by presenting an integrative framework. It connects panoramic retinal assessment, AI-driven multimodal analysis, and potential systemic risk stratification. While each of these elements has been studied separately, their interplay in the context of UWF-CFP-based DR management has not been fully examined, and this review aims to provide a structured overview of that intersection. Through the connection between peripheral lesion detection, AI-enhanced quantitative vascular parameters, and non-invasive assessment of possible systemic complications (such as diabetic nephropathy, cardiovascular diseases, and stroke), this review provides a clinically-oriented perspective that may help guide the comprehensive management of patients with type 2 diabetes (T2DM). It should be noted, however, that the proposed “eye–system collaborative management” framework remains conceptual and requires rigorous prospective evaluation before clinical adoption. In this context, we tentatively propose the concept of “eye-system collaborative management.” This structured care model integrates retinal microvascular parameters from UWF-CFP with systemic clinical data. It thereby enables non-invasive risk assessment of diabetic complications (e.g., nephropathy, cardiovascular disease, and stroke) and guides multidisciplinary coordination among ophthalmologists, endocrinologists, nephrologists, and cardiologists.

This review focuses exclusively on adult patients (≥18 years) with T2DM. Evidence pertaining to children, adolescents, or pregnant women with diabetes is not covered, as the epidemiology, screening strategies, and intervention thresholds in these populations differ substantially and remain insufficiently studied for UWF-CFP. Specifically, we address three key questions: (1) How does UWF-CFP overcome the limitations of traditional ETDRS 7-field photography and refine the staging system for DR through the identification of peripheral lesions? (2) How does the integration of UWF-CFP with deep learning algorithms and multimodal imaging improve the accuracy of automated screening, precise grading, and quantitative vascular analysis? (3) Can the retinal microvascular parameters captured by UWF-CFP serve as non-invasive biomarkers for diabetes-related complications, thereby enabling the “eye-system collaborative management” model? By integrating existing evidence and identifying existing gaps, this review aims to explore the clinical translation direction and future research focus of UWF-CFP in the era of precision medicine.

## 2 Methods

This review was designed as a narrative review with a systematic search strategy, and the synthesis is qualitative rather than quantitative. PubMed and Web of Science were searched up to May 2, 2026. The search strategy combined terms related to ultra-widefield imaging (“ultra-widefield color fundus photography,” “UWF-CFP,” “ultra-widefield imaging,” “ultra-widefield fundus photography” “ultrawidefield imaging,” “ultrawidefield fundus photography”) using the OR operator, and combined them with diabetic retinopathy (“diabetic retinopathy,” “DR”) using the AND operator. Additional searches were performed for artificial intelligence (“deep learning,” “AI,” “machine learning,” “convolutional neural network,” “CNN,” “automated detection,” “automated grading”) and systemic complications (“diabetic kidney disease,” “chronic kidney disease,” “cardiovascular disease,” “cardiovascular risk,” “stroke,” “cerebrovascular disease,” “systemic disease”).

Inclusion criteria:

(1) Study design: Original research (including prospective/retrospective cohort studies, cross-sectional studies, case-control studies, and diagnostic accuracy studies), clinical trials (randomized or non-randomized), and systematic reviews (with or without meta-analysis).
(2) Outcomes: Studies must address at least one of the following:
① DR assessment: DR screening, severity grading (e.g., mild/moderate/severe NPDR, PDR), detection of peripheral lesions (e.g., predominantly peripheral lesions, peripheral non-perfusion, neovascularization elsewhere), or DR progression.
② AI-based analysis: Development or validation of deep learning/machine learning models for automated DR detection, lesion segmentation, quantitative vascular analysis, or multimodal integration.
③ Systemic complications: Correlation of UWF-CFP-derived retinal parameters with diabetic kidney disease, cardiovascular disease, or cerebrovascular disease (e.g., stroke).
(3) Population: Studies involving adult patients (≥18 years) with T2DM.
(4) Language: English.

Exclusion criteria:

(1) Publication type: Conference abstracts, case reports (≤5 cases), editorials, letters, commentaries without original data.
(2) Lack of relevant outcomes: Studies focusing exclusively on diabetic macular edema without providing DR severity grading.
(3) Population: Studies focusing exclusively on type 1 diabetes, gestational diabetes, or pediatric populations were excluded.
(4) Full-text unavailable: Articles for which the full text cannot be obtained after reasonable effort.

The same criteria were applied during both title/abstract screening and full-text assessment. Any study that met all inclusion criteria and none of the exclusion criteria was eligible, regardless of its findings.

**FIGURE 2:**
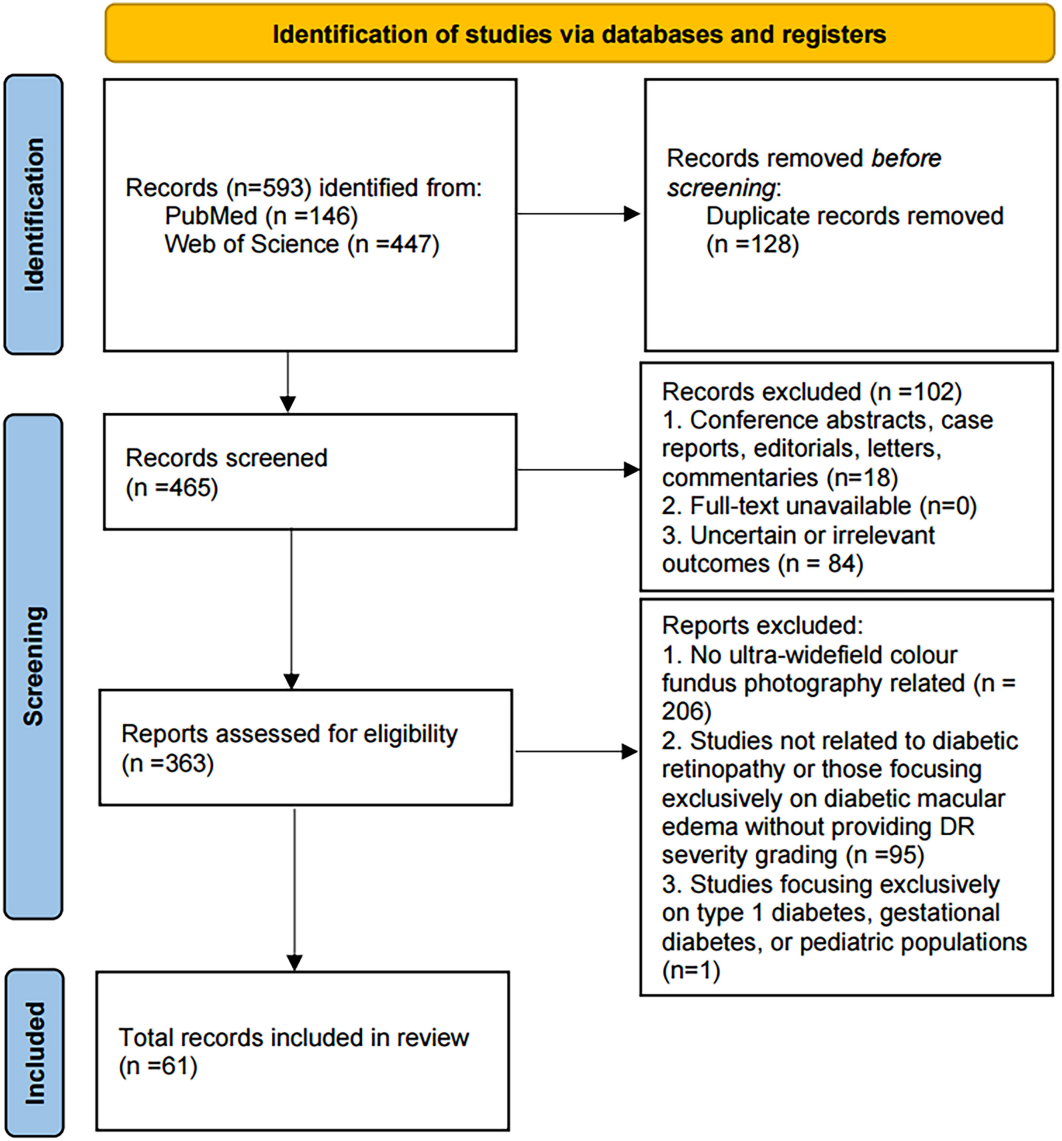
PRISMA 2020 flow diagram of study selection. A total of 593 records were identified from PubMed (*n* = 146) and Web of Science (*n* = 447). After removal of duplicates (*n* = 128), 465 records were screened. At the title/abstract screening stage, 102 records were excluded: 18 were conference abstracts, case reports, editorials, letters, or commentaries; full-text was unavailable for 0 records; and 84 records were excluded due to uncertain or irrelevant outcomes. The remaining 363 full-text reports were assessed for eligibility. Of these, 206 reports were excluded because they were not related to ultra-widefield color fundus photography; 95 reports were excluded because they were either not related to diabetic retinopathy (DR) or focused exclusively on diabetic macular edema without providing DR severity grading; and 1 report was excluded for focusing exclusively on type 1 diabetes, gestational diabetes, or pediatric populations. Ultimately, 61 studies were included in the review. The figure provides a transparent and reproducible summary of the screening process, highlighting the focused scope of the review and the key inclusion criteria.

Two reviewers (R.F. and R.X.) independently screened titles, abstracts, and full texts against the predefined criteria. Disagreements were settled by consensus or a third reviewer (Y.T.). Manual screening of reference lists from included articles was performed to identify additional relevant studies. The study selection process is summarized in Figure 2, which was adapted from the PRISMA 2020 flow diagram to enhance transparency. Figure 2 depicts the entire filtering cascade from the initial identification to the final inclusion. The legend provides detailed explanations for the specific reasons for each screening stage’s exclusions and how these exclusions define the boundaries of the evidence base.

Because this is a narrative review rather than a systematic review, formal risk-of-bias tools designed for systematic reviews (e.g., ROBIS, AMSTAR) were not applicable. Instead, a simplified qualitative assessment of the included studies was performed. Studies were categorized by design type: prospective cohort studies and diagnostic accuracy studies were regarded as providing a relatively higher level of evidence, whereas cross-sectional, retrospective, and case-control studies were considered more susceptible to selection bias and unmeasured confounding. Among the 61 included studies, a small proportion were of prospective cohort or diagnostic accuracy design or prospective observational study, and the majority were cross-sectional or retrospective analyses or case-control studies. This imbalance is acknowledged as a limitation of the current evidence base and is discussed further in Section “6.5 Quantitative trend interpretation and its evidence boundaries.” No study was excluded solely on the basis of design, but design type was taken into account when interpreting the strength of evidence. The remaining studies comprised systematic reviews, or narrative reviews, which were used for background and discussion but were not assessed as original evidence. The Newcastle-Ottawa Scale quality assessment scores for individual studies are presented in Supplementary Figure 1. An overview of the design, sample size, imaging device, key findings, and level of evidence for the main studies included in this review is presented in Table 2.

**TABLE 2:** Characteristics and key findings of studies included in this review.

References	Study design	Sample size (patients/ eyes)	Imaging device	Primary outcome(s)	Key finding with effect size	Level of evidence*
Silva et al. (9)	Prospective cohort	206 eyes	Optos^®^	Distribution of PPL and DR severity	Approximately one-third of H/Ma, IRMA, and NVE were predominantly outside ETDRS fields; UWF -CFP suggested more severe DR in 10% of eyes.	II
Silva et al. (37)	Prospective cohort	200 eyes	Optos^®^	PPL and risk of DR progression/PDR development	Eyes with PPL had a 3.2-fold higher risk of DR progression and a 4.7-fold higher risk of progression to PDR; approximately half of DR eyes had PPL outside ETDRS 7-field	II
Aiello et al. (11)	Multicenter cross-sectional	764 eyes	Optos^®^	Agreement between UWF-CFP and ETDRS 7-field	High agreement in DR grading; UWF-CFP detected more peripheral lesions: PPL present in 308 of 751 eyes (41.0%), suggesting increased DR severity by ≥2 steps in 34 eyes (11.0%).	III
Marcus et al. (29)	Prospective cohort	544 eyes	Optos^®^	PPL and risk of DR progression	PPL associated with 1.7-fold increased risk of DR worsening over 4 years	II
Verma et al. (10)	Retrospective study	1406 eyes	Optos^®^	Distribution of DR lesions (PPL vs. PCL) across ICDR severity grades in an Indian population	PPL distribution in 37% of eyes vs. PCL in 63% (*P* < 0.003); PPL frequency varied by ICDR grade: mild NPDR 30.9%, moderate NPDR 40.3%, severe NPDR 38.5%, PDR 34.9% (*P* = 0.005); temporal fields showed greatest PPL frequency across all grades	II
Silva et al. (38)	Retrospective study	544 eyes	Optos^®^	Effect of DR lesion type, severity, and distribution on disease worsening (≥2-step ETDRS-SS worsening or DR treatment over 4 years)	More severe lesion grades outside ETDRS fields associated with greater risk of disease worsening: on UWF-CFP, H/Ma HR 1.74 (1.28–2.36); on UWFFA, H/Ma HR 1.90 (1.38–2.61), IRMA HR 1.68 (1.13–2.49), NVE HR 1.99 (1.36–2.93)	III
Stino et al. (30)	Cross-sectional	75 eyes	Optos^®^ + UWFFA	Correlation between IRMA and NPA	Number of IRMA regions highly correlated with NPA area; r_*s*_ = 0.86 (*P* < 0.01)	III
Li et al. (34)	Prospective cohort	153 eyes	Optos^®^ + UWF SS-OCTA	UWF-CFP + UWF SS-OCTA vs. UWF-CFP + FFA for DR grading	Very good agreement in DR severity; κ = 0.869; combined non-invasive approach showed similar lesion detection rates to FFA (*p* > 0.05 for all, except NPA *p* = 0.039)	II
Cui et al. (35)	Prospective observational study	152 eyes	Optos^®^ + UWF SS-OCTA	UWF-CFP + UWF SS-OCTA vs. UWFFA for DR lesion detection rate	Combined UWF SS-OCTA + UWF-CFP achieved equivalent DR lesion detection rates to UWFFA (*p* > 0.05), with high DR grading agreement (κ = 0.916)	II
Zhao et al. (40)	Multicenter diagnostic	59,475 images	Optos^®^	Deep learning model (WARM) for 25 fundus diseases	WARM superior to region-restricted model for peripheral/diffuse lesions	II
Deng et al. (43)	Cross-sectional/AI	339 patients	Optos^®^	Quantitative vascular analysis (fractal dimension, density)	Vascular density and fractal dimension decreased with increasing DR severity; fractal dimension: 1.33 ± 0.05 vs. 1.41 ± 0.03; vascular density: 1.12 ± 0.44 vs. 2.09 ± 0.36	III
Rezaei et al. (20)	Cross-sectional/AI	8,524 images from BRSET + 5,164 images from mBRSET	Zeiss Clarus^®^	UWF-CFP + clinical data vs. images alone	Multimodal model improved accuracy by 2.1% and Kappa by 0.022 on primary dataset; external validation Kappa improvements of 0.065 and 0.048	III
Meng et al. (54)	Cross-sectional/AI	734,084 images	Conventional fundus camera	DeepDKD model for DKD prediction	DeepDKD AUC 0.842–0.906; image model significantly superior to metadata model (AUC 0.680); combined model AUC 0.847; prospective validation sensitivity: combined model 86.7% vs. image-only 89.8% vs. metadata-only 66.3%enabled non-invasive DKD screening	II
Vujosevic et al. (55)	Multicenter cross-sectional	516 eyes	UWF-CFP (Optos^®^) + OCT + OCTA	DR severity and history of stroke	Each 1-step increase in DR severity associated with 47% higher stroke risk; OR 1.47 (95% CI 1.02–2.11, *p* = 0.044); middle capillary plexus perfusion density (*p* = 0.017) and vessel density (*p* = 0.010) most significantly associated with stroke history each 1-step increase in DR severity: OR for stroke = 1.47 (95% CI: 1.02–2.11)	III
Xu et al. (41)	Generative AI/cross-sectional	270 paired UWF-CFP and UWF-FA images	Optos^®^	GAN-synthesized UWF-FA from UWF-CFP	UWFDR-GAN outperformed all comparison models: MS-SSIM 0.7214; PSNR 20.00; FID 77.48; IS 1.0123. Qualitatively superior reconstruction of neovascularization and non-perfusion areas, with preserved global vascular architecture. Demonstrated potential to reduce reliance on invasive fluorescein imaging synthetic FA aided DR grading without intravenous dye	III
Abitbol et al. (12)	Retrospective study	224 images	Optos^®^	Deep learning for retinal vascular disease classification	Per-class performance: DR AUC 90.5%, accuracy 85.2%. Cross-device generalizability not validated high performance but cross-device generalizability not fully validated	III
Ploumi et al. (56)	Cross-sectional	182 eyes	UWF-CFP	Association between PPL and systemic complications	PPL significantly associated with peripheral arterial disease (OR 11.36, *p* = 0.033), coronary artery disease (OR 5.86, *p* < 0.001), stroke (OR 10.11, *p* = 0.003), and diabetic nephropathy (OR 2.97, *p* = 0.016); eGFR decreased with PPL presence (β = −12.66, *p* = 0.037)	III

*Level of evidence: II = prospective cohort/diagnostic accuracy study/prospective observational study; III = cross-sectional/retrospective/case-control study (non-randomized). Only original studies are listed; reviews and commentaries are excluded. This classification is intended as a qualitative guide and does not imply formal risk-of-bias assessment. OR, odds ratio; HR, hazard ratio; κ, Cohen’s kappa; r_s, Spearman’s rank correlation coefficient; AUC, area under the curve; CI, confidence interval. H/Ma, hemorrhages and/or microaneurysms; IRMA, intraretinal microvascular abnormalities; NVE, neovascularization elsewhere; NPA, non-perfusion area; PPL, predominantly peripheral lesions; PDR, proliferative diabetic retinopathy; NPDR, non-proliferative diabetic retinopathy; DKD, diabetic kidney disease; CAD, coronary artery disease; PAD, peripheral arterial disease; eGFR, estimated glomerular filtration rate.

## 3 UWF-CFP in the diagnosis and staging of DR

### 3.1 Assessment of peripheral non-perfusion and identification of specific lesions

Peripheral retinal non-perfusion area (NPA) underpins the progression of DR from non-proliferative to proliferative stages; therefore, quantifying NPAs in DR is of critical importance (23). Fundus fluorescein angiography (FFA) has long been the gold standard for evaluating NPAs. Ultra-widefield fluorescein angiography (UWFFA) is a dynamic vascular imaging technique acquired through intravenous sodium fluorescein injection using the UWF-CFP platform. It dynamically captures the dye filling process and depicts NPAs as characteristic dark areas (24). In terms of identifying neovascularization and intraretinal microvascular abnormalities (IRMA), the dynamic imaging of UWFFA clearly demonstrates the characteristic leakage patterns of neovascularization, which typically exhibits marked fluorescein leakage with blurred margins in the late phase (25); in contrast, although IRMA represents abnormal vascular channels, it usually shows no or only minimal leakage. This distinction is critical for differentiating severe non-proliferative diabetic retinopathy (NPDR) from proliferative diabetic retinopathy (PDR) (26). However, the invasive nature, time-consuming procedure, and potential risk of contrast agent allergy limit the application of FFA in routine screening and frequent follow-up (27). Therefore, exploring non-invasive techniques for assessing peripheral NPAs has become a research hotspot in DR imaging.

Although UWF-CFP lacks functional contrast and cannot directly visualize NPAs, studies have found that certain morphological changes visible on UWF-CFP are closely associated with NPAs. Predominantly peripheral lesions (PPL) serve as an important clinical clue suggesting extensive peripheral non-perfusion and independently influence DR severity grading (28). The detection of PPL on UWFFA has been shown to be associated with a 1.7-fold increased risk of DR progression over 4 years (29). Furthermore, the distribution, number, and severity of various DR characteristic lesions located in the peripheral retina are closely correlated with the degree of underlying ischemia. The number of regions affected by IRMA on UWF-CFP is highly correlated with the area of non-perfusion (rs = 0.86, *P* < 0.01), and the distribution of lesions such as microaneurysms and hemorrhages shows significant topographic overlap with underlying non-perfused areas (30).

In terms of identifying specific lesions, UWF-CFP plays an irreplaceable role in differential diagnosis and treatment decision-making due to its wide field of view and high-resolution imaging. Retinal neovascularization, a hallmark lesion of PDR, is categorized by location as neovascularization of the optic disc (NVD) or elsewhere (NVE) (31, 32). With its 200° ultra-widefield view, UWF-CFP may detect NVE located in the far peripheral retina that are frequently missed by conventional seven-field photography. However, in the absence of angiographic confirmation, differentiating between small NVE and IRMA remains a challenge (33). However, UWF-CFP alone often cannot reliably distinguish early peripheral neovascularization from IRMA (34). Combining UWF-CFP with ultra-widefield swept-source optical coherence tomography angiography (UWF SS-OCTA) can help overcome this limitation (35). Research indicates that combining UWF-CFP with UWF SS-OCTA shows no significant differences in lesion detection rates such as microaneurysms, intraretinal hemorrhages, IRMA, venous beading, and neovascularization compared with UWF-CFP combined with FFA; specifically, sensitivity for NVE is 92.3% and specificity 96.8%, while for IRMA sensitivity is 88.9%, specificity 93.5% (κ = 0.823), with high consistency in DR severity grading (κ = 0.869) (34). Combining UWF SS-OCTA with UWF-CFP offers a promising non-invasive alternative to UWFFA for DR assessment. However, it should be noted that the strength of the correlation between IRMA and NPA varies among different research reports (30, 34). These differences may reflect variations in image acquisition protocols, peripheral area definitions, and the inherent subjectivity in the IRMA grading. Before adopting a standardized quantitative framework, the universality of these findings remains uncertain.

### 3.2 Contributions and implications of UWF-CFP for DR staging systems

**FIGURE 3:**
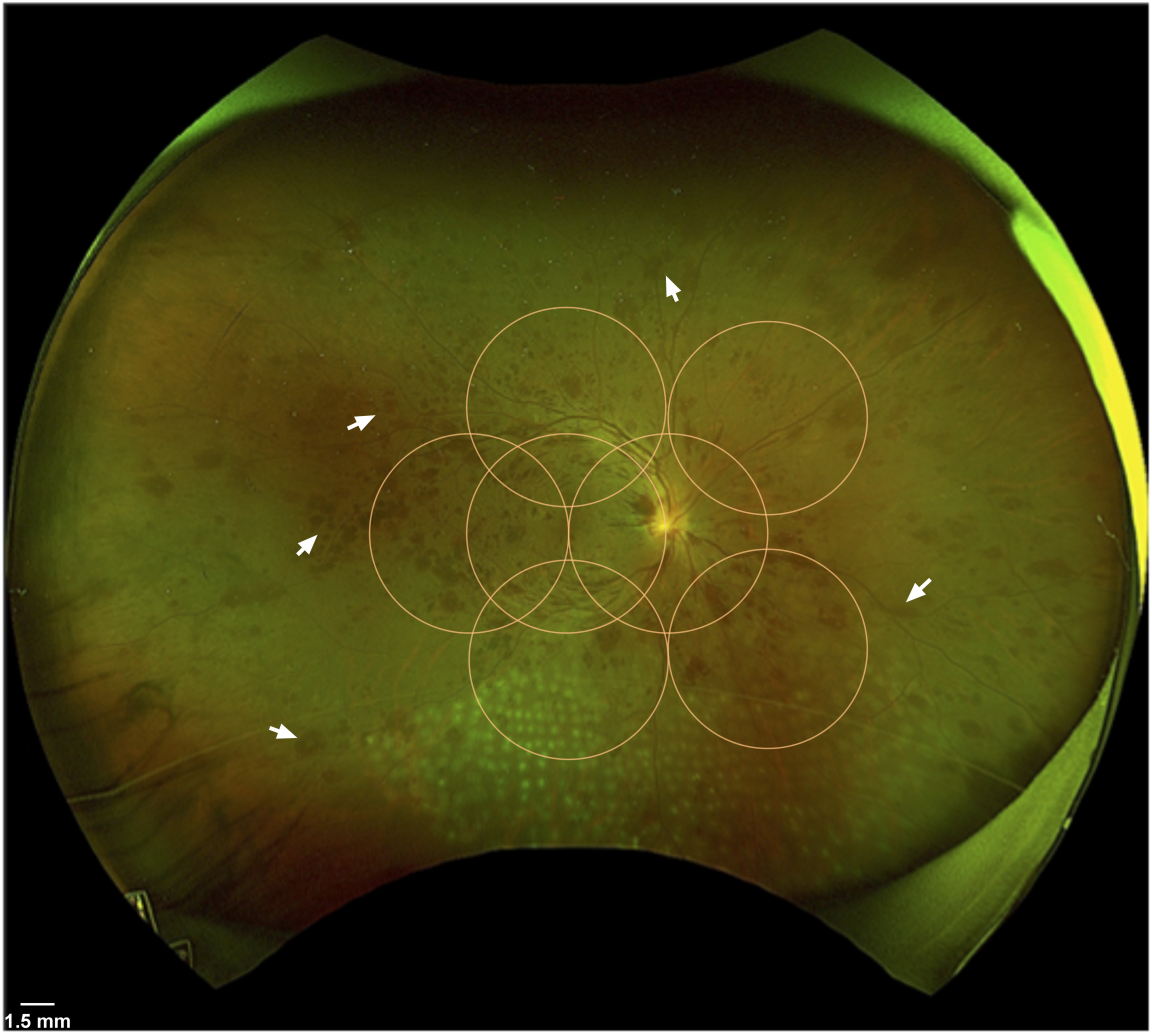
Ultra-widefield color fundus photography (UWF-CFP) with the Optos^®^ system demonstrating peripheral lesions in diabetic retinopathy (DR) beyond the coverage of ETDRS 7-field photography. The seven circles indicate the standard positions and extents of ETDRS 7-field photography (each field covers 30°, collectively covering approximately 35% of the retina). Solid white arrows mark peripheral lesions located outside the circle (including microaneurysms, hemorrhage and retinal microvascular abnormalities) represent peripheral dominant lesions. These lesions may be overlooked by traditional imaging methods, leading to an underestimation of the severity of DR. The scale bar in the lower left corner represents 1.5 mm.

With the widespread clinical application of UWF-CFP, the criteria for DR staging are shifting from ETDRS 7-field grading toward a panretinal evaluation approach. Multiple studies have confirmed that UWF-CFP shows high consistency with ETDRS seven-field photography in determining DR severity grading, while offering higher detection rates (7, 36). Nevertheless, these studies differ in the grading protocols used and in how peripheral fields are defined, which limits direct comparability of the reported increments. The most significant contribution of UWF-CFP to the DR staging system lies in revealing the high prevalence of PPL and their clinical significance. According to a prospective cohort study (*n* = 200 eyes), eyes with PPL had a 3.2-fold higher risk of DR progression and a 4.7-fold higher risk of progression to PDR. Approximately half of DR eyes presented with lesions situated primarily outside the ETDRS seven-field protocol (37). These estimates, while clinically meaningful, were derived from a single study with a predominantly Caucasian population and should be validated in independent, multi-ethnic cohorts. This indicates that a considerable number of patients would have their disease severity systematically underestimated if staging were based solely on posterior pole images (38). Figure 3 provides direct visual evidence for this argument. The ETDRS 7-field circles are superimposed as a spatial reference, demarcating the boundary between conventionally assessed and unassessed retinal territory. A detailed annotation of the visible peripheral lesions and their clinical significance is provided in the figure legend. Outside the seven circles representing the ETDRS fields, extensive PPL–including microaneurysms, hemorrhages, IRMA, and peripheral non-perfusion areas–are clearly visible. Such findings directly challenge the completeness of traditional staging systems. Accordingly, some scholars have proposed expanding the retinal assessment range for DR from the conventional 30° to over 200° and incorporating PPL into the staging framework. In the future, with the increasing availability of UWF-CFP devices and the maturation of AI-assisted quantitative tools, a new DR staging system based on panretinal assessment will gradually be refined, enabling a shift from “seeing locally” to “insight into the whole.”

The PPL detected on UWF-CFP has direct guiding significance for clinical management. Patients with PPL have a significantly increased risk of DR progression and proliferative lesions, suggesting that such findings should prompt more intensive monitoring and earlier intervention. Additionally, if extensive peripheral lesions are found, especially IRMA or suspicious peripheral neovascularization, the threshold for performing UWFFA should be lowered to identify non-perfused areas or active neovascularization that require laser photocoagulation or anti-VEGF treatment. Although these adjustments for clinical practice are conceptually reasonable, they still need to be prospectively validated before being incorporated into formal guidelines.

## 4 Artificial intelligence models based on UWF-CFP and deep learning in DR

Recently, research on AI applications in DR has been gaining considerable momentum. A bibliometric-based systematic analysis revealed that automated screening and grading of DR have become research hotspots in this field, with increasing attention being paid to algorithm generalizability and multicenter validation (39). Building on this foundation, deep learning models based on UWF-CFP have also made significant progress. A growing body of work has demonstrated that deep learning algorithms can achieve high accuracy in detecting DR from UWF-CFP images and grading disease severity, though most studies have used internal validation only and reported performance on single-device datasets, which may overestimate real-world generalizability (20, 40). One multicenter study constructed a large-scale, high-quality dataset containing approximately 60,000 UWF-CFP images. Based on the Swin Transformer architecture and an original cross-domain collaborative learning algorithm, developed three deep learning models–Wide-range lesion Recognition Model (WARM), Baseline Model (BASE), and WARM with Peripheral Performance Restriction (WARM-PPR)–to accurately identify 25 fundus diseases, including DR (40). This multicenter validation demonstrated that the WARM model achieved significantly better performance in identifying peripheral and diffuse lesions compared with the region-restricted WARM-PPR model. Another study matched UWF-CFP with UWFFA images to train a model based on the pix2pixHD generative adversarial network (GAN) and validated the contribution of the synthesized UWF-FA images to DR grading. The study demonstrated that a GAN-based model can generate realistic multi-frame UWF-FA images from UWF-CFP, thus enhancing DR grading without intravenous dye injection (41). Furthermore, studies have shown that, compared with manual reading of UWF-CFP images, AI-based reading achieves a favorable balance between sensitivity and specificity, and when AI serves as an assistant, it significantly improves the accuracy of DR diagnosis and grading (42). However, most of the validations have been conducted through retrospective studies in large datasets, and there is still a lack of prospective real-world evidence.

In the field of automated quantitative analysis of UWF-CFP images, deep learning technology has demonstrated the ability to precisely segment key pathological features. Quantitative vascular measurements based on UWF-CFP include indicators such as vascular diameter, tortuosity, and fractal dimension, which are key indicators for predicting the progression of DR. A study used RU-net combined with transfer learning to segment the vessels in UWF-CFP images, achieving an accuracy rate of 99% and a Dice coefficient of 0.76. Based on this study, it was found that the vascular angles of patients in the DR group were smaller (33.68 ± 3.01 vs. 37.78 ± 1.60), the fractal dimension was lower (1.33 ± 0.05 vs. 1.41 ± 0.03), the vascular density was smaller (1.12 ± 0.44 vs. 2.09 ± 0.36), and the number of vascular branches was fewer (206.1 ± 88.8 vs. 396.5 ± 91.3). Additionally, as the severity of DR increased, the fractal dimension value showed a downward trend (43). These quantitative indicators provide an objective basis for the early diagnosis and disease monitoring of DR.

Although deep learning models have achieved high accuracy in controlled research environments, their generalization ability in different clinical settings remains a significant challenge. Currently, there is still a lack of direct head-to-head comparisons among the deep learning models applicable to UWF-CFP. Most of the published models were developed on single-device datasets and evaluated only using the internal test set, resulting in potentially optimistic performance estimates. External validation, especially when the test device or population differs from the training data, often results in performance decline. Thus, the current literature does not support a clear conclusion regarding algorithmic superiority in UWF-CFP-based DR analysis. The Optos^®^ series uses a confocal scanning laser fundus camera with pseudo-color imaging, while the Zeiss Clarus^®^ system employs multispectral true color technology and has a narrower field of view. These differences result in significant changes in image appearance, color representation, and artifact patterns. If no adaptive adjustments are made, the performance of the model can be substantially affected when applied to data from different devices (44). Similarly, cross-population generalization has not been fully explored; most training datasets come from specific racial groups, raising concerns about the universality of the model’s performance in diverse populations with different retinal pigmentation and disease phenotypes (45). Moreover, the ability of models trained only for DR to generalize to other retinal vascular diseases (such as retinal vein occlusion or hypertensive retinopathy) has not been systematically evaluated. To address these gaps, multi-center prospective validation (46), improved domain adaptation techniques, and the development of diverse, publicly accessible UWF-CFP datasets covering different devices, races, and disease spectra are needed.

## 5 Multimodal integration based on UWF-CFP for comprehensive DR assessment

**FIGURE 4:**
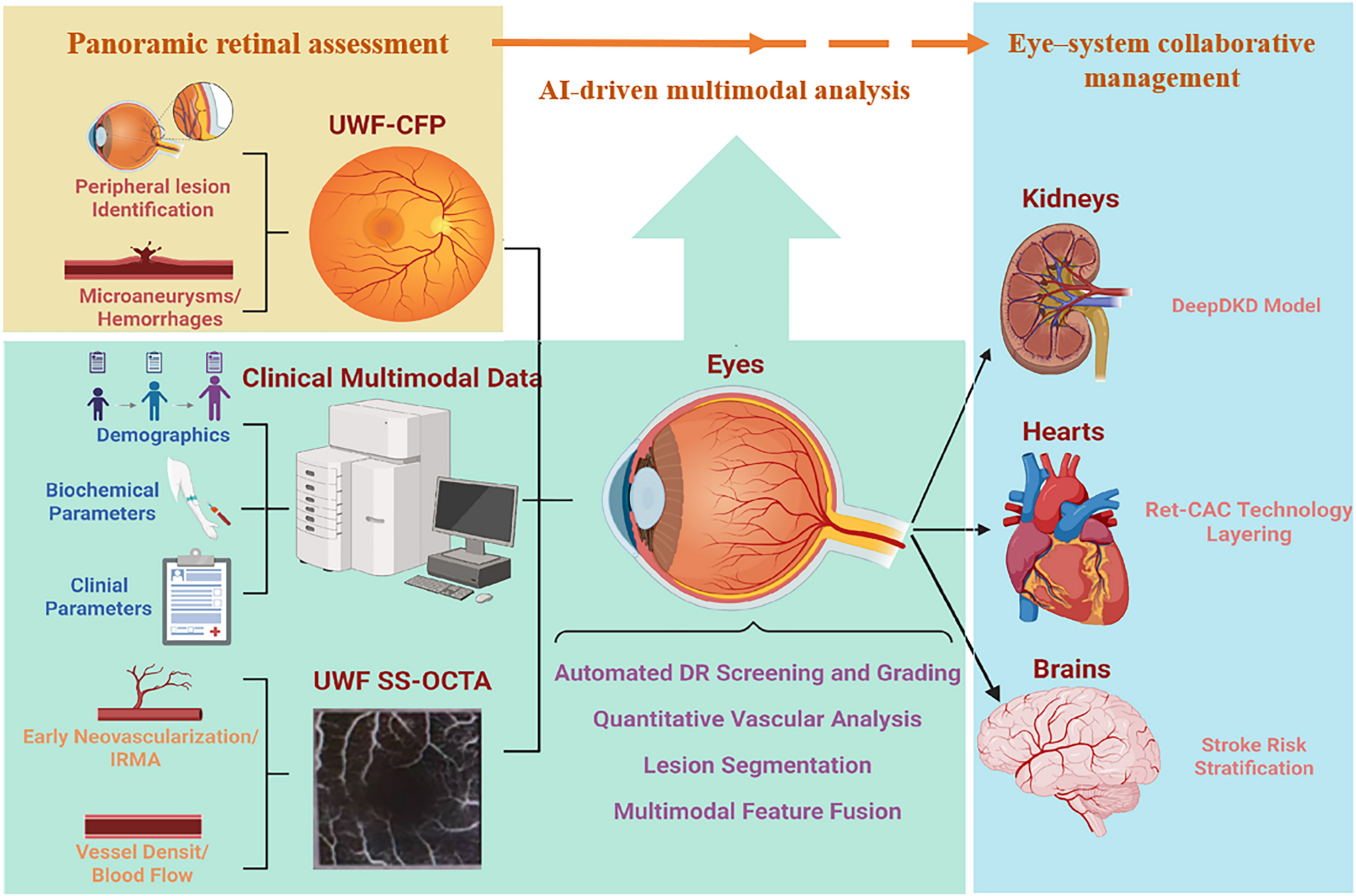
Multimodal analysis integration framework based on UWF-CFP. The three-section structure illustrates the progressive evolution of DR management. Panoramic Retinal Assessment (yellow section): with UWF-CFP as the core imaging method, it comprehensively presents peripheral lesions, microaneurysms and hemorrhage that are often missed in traditional ETDRS 7 field photography, laying the foundation for assessment; AI-driven Multimodal Analysis (green section): integrating clinical multimodal data (demographics, biochemical parameters, clinical parameters) with UWF SS-OCTA images, achieving automatic screening classification, quantitative vascular analysis, lesion segmentation, and multi-modal feature fusion; Eye–system Collaborative Management (blue section): extending imaging biomarkers to systemic risk stratification (diabetic kidney disease, coronary artery calcification, stroke risk assessment). Arrows indicate the progressive pathway from retinal assessment toward systemic management. The dashed arrow in the blue section indicates that “eye–system collaborative management” remains a conceptual framework requiring prospective validation. UWF-CFP, ultra-widefield color fundus photography; UWF SS-OCTA, ultra-widefield swept-source optical coherence tomography angiography; IRMA, intraretinal microvascular abnormalities; DR, diabetic retinopathy. Created in BioRender. Fang, R. (2026) https://BioRender.com/qzzwo95.

The severity of DR is not only influenced by the characteristics of fundus imaging, but also by systemic clinical variables. The multimodal integration described in this chapter (as outlined in Figure 4) refers to combining complementary data streams such as UWF-CFP images, OCTA images, and structured clinical parameters within a unified analysis framework to achieve a more comprehensive and accurate assessment (20, 34). This chapter reviews the current status of multimodal integration with UWF-CFP as the core imaging modality, covering UWF-CFP and OCTA integration, UWF-CFP and structured clinical data integration, and the extension of system disease risk prediction, while identifying the key methodological gaps that still need to be addressed.

### 5.1 Integration of UWF-CFP and OCTA for peripheral lesion characterization

The current research mainly focuses on integrating UWF-CFP with high-resolution modalities such as OCTA to meet the clinical needs related to the detection of peripheral lesions and the fine assessment of microvasculature. From the perspective of structural correlation, the integration of UWF-CFP and OCTA addresses the complementary requirements of panoramic coverage and deep resolution of microvascular details. UWF-CFP can display the distribution of peripheral lesions within a 200° range, but it cannot reliably distinguish IRMA from early neovascularization, nor can it identify areas without perfusion. OCTA, on the other hand, provides a three-dimensional vascular mapping through depth-encoded blood flow signals. In clinical studies, Li et al. (153 eyes) and Cui et al. (152 eyes) adopted a common multimodal workflow. At the front-end input, both studies collected images using a unified approach on the same day, automatically segmented the vascular layers using the built-in software of the device, and manually corrected the errors at the vitreoretinal interface. During automatic vessel layer segmentation, manual correction was also applied. Two blinded assessors independently reviewed the images. When disagreements occurred, a third party was consulted for arbitration. Two studies jointly confirmed that the detection rate of DR lesions by UWF CFP combined with UWF SS-OCTA was completely equivalent to that by UWF CFP combined with FFA/UWFFA (both *p* > 0.05), and the severity grading of DR achieved very good consistency (κ = 0.869^34^; κ = 0.916^35^). On the deep learning side, the same structural complementarity is transformed into a two-stream network architecture. In the front-end input, 2D planar and 3D volumetric data are preprocessed with modality-specific methods. ResNet50 and 3D-ResNet50 are used to extract their respective features, and the Squeeze-and-Excitation module is inserted for adaptive feature calibration. The features are then fused using an intermediate hierarchical strategy at the back-end, and a manifold mixing strategy is introduced to alleviate overfitting caused by limited paired data (47). Although this computational study is still preliminary, the design logic of its front-end differentiated processing, complementary feature extraction, and back-end fusion is consistent with the integration principles established in clinical literature. The results showed that compared with the single-modal benchmark model, the DR classification performance of this fusion framework was significantly improved (46).

In terms of resolution compensation, UWF-CFP has a wide spatial coverage but lacks depth resolution, whereas UWF SS-OCTA provides high-precision three-dimensional vascular information within a narrower field of view. A cross-sectional study involving 90 eyes used Optos California to obtain UWF-CFP and UWFFA, and Toward Pi BM400K to obtain UWF SS-OCTA. The results showed that the sensitivity of UWF SS-OCTA for detecting new blood vessels was 0.97 and the specificity was 1.0. The count of new blood vessels within a 24 mm × 20 mm field of view was highly consistent with UWFFA (ICC = 0.994). After combined panoramic assessment with UWF-CFP, the detection consistency further improved (ICC = 0.988, 95% CI: 0.975–0.994), confirming the synergistic pattern of spatial positioning provided by UWF-CFP and confirmation of blood flow structure provided by OCTA (48). These findings collectively indicate that multimodal integration can effectively balance field of view and resolution, and combine panoramic screening with deep lesion characterization.

### 5.2 Integration of UWF-CFP with structured clinical data for severity assessment

The severity of DR is not only determined by the imaging manifestations of the fundus, but is also closely related to clinical factors such as disease duration, blood glucose control level, blood pressure and blood lipids. A risk prediction model based solely on non-imaging clinical features (such as disease duration, blood glucose, blood pressure, etc.) successfully achieved effective stratification of vision-threatening DR, highlighting the potential of multi-source data integration for precise DR assessment (49). From the perspective of information complementarity, the integration of UWF-CFP with structured clinical data has bridged the information gap between structural imaging findings and the overall metabolic status. Deman et al. further proposed a multimodal deep learning framework. It combines UWF-CFP images with 76 clinical features (including demographics, biochemical indicators and clinical parameters) for automatic DR severity assessment. Methodologically, this study adopted a patient-level data partition strategy at the input stage to avoid the inflated performance caused by random image division. The image branch was enhanced through random cropping, scaling, and color jittering; the table branch was processed with missing value filling, ordered encoding, and normalization. In the feature extraction stage, the ImageNet pre-trained ResNet50 was used as the backbone network for image feature extraction, and various table classifiers such as MLP, FT-Transformer, GANDALF, and TabPFN were evaluated. The features of the two modalities were projected to a unified intermediate dimension, concatenated, and input to the classification layer. The MLP-ResNet50 combination achieved the best overall performance. On two independent Brazilian external datasets (BRSET and mBRset), the multimodal fusion model consistently outperformed the pure image single-modal classifier, with Kappa values increasing by 0.065 and 0.048, respectively. Compared with a unimodal classifier using images alone, this approach improved accuracy by 2.1% and the Kappa value by 0.022 (20). This shows that the fusion of clinical data can provide supplementary information for imaging assessment, but the overall improvement is relatively limited.

However, this study only relied on traditional ImageNet supervised pre-training initialization and did not adopt self-supervised pre-training strategies. The dataset came from a single device and a single population. It did not adopt domain adaptation, federated learning, or large base model transfer strategies, which limited the model’s generalization ability in different ultra-wide-angle imaging devices and different racial populations. Moreover, the external validation datasets used conventional fundus photography rather than UWF-CFP. This precludes direct cross-dataset performance comparisons. In the future, more prospective studies with multiple devices and multiple populations need to be conducted to address these deficiencies.

### 5.3 Extension of UWF-CFP-based multimodal models to systemic complication risk prediction

From the perspective of structural correlation, the theoretical basis for extending the application scope of multimodal fusion from ocular lesion assessment to the prediction of systemic complication risks lies in the homology of the pathological physiological mechanisms between retinal microcirculation and microcirculation in the kidneys, heart, and brain (50–53). At the computational level, Meng et al.’s DeepDKD study, although based on traditional fundus color photographs, provided a transferable methodological framework. In terms of the front-end input, it was compatible with various standard fundus cameras and structured clinical data. The back-end used self-supervised pre-trained ResNet-50 encoding, and the XGBoost was used to integrate the images and metadata at the decision-making level. The results showed that the pure image model (AUC 0.842) was significantly better than the pure metadata model (AUC 0.680), and the fusion model achieved an AUC of 0.847. This suggests that if UWF-CFP replaces traditional color photographs, the fusion should shift from the later decision-making layer to the intermediate feature layer to fully exploit the incremental value of peripheral microvascular information (54). In the clinical validation aspect, there were two studies directly conducted under the UWF-CFP framework. Vujosevic et al.’s multicenter cross-sectional study integrated UWF-CFP’s DR grading and PPL assessment, OCTA microvascular quantitative indicators, and clinical variables. It found that for each increase in DR severity, the risk of previous stroke increased by 47% (OR = 1.47). The middle capillary plexus perfusion density and vascular density were the most significant parameters related to stroke history (55). Another cross-sectional study, which included 182 eyes, collected and independently evaluated PPL using standardized UWF-CFP at the front end. The back-end associated PPL with systematic complication data extracted from electronic medical records, finding that the presence of PPL was significantly associated with peripheral arterial disease (OR = 11.36), coronary heart disease (OR = 5.86), and stroke (OR = 10.11), and was also significantly associated with diabetic nephropathy (OR = 2.97) and a decrease in eGFR (β = −12.66) (56). These two studies formed a complementary methodology, jointly suggesting that the peripheral lesion information on UWF-CFP may carry systemic risk stratification value. These findings offer preliminary support for the hypothesis that integrating UWF-CFP and OCTA feature branches within an AI multimodal system could enhance risk assessment, though this remains to be confirmed in prospective studies. Additionally, a conference abstract preliminarily reported that Ret-CAC screening based on traditional fundus photography can detect coronary artery calcification, but large-scale prospective studies are still needed for verification (57).

In terms of resolution compensation, the aforementioned research has revealed a hierarchical complementary logic. UWF-CFP provides the macroscopic spatial distribution of peripheral lesions (represented by PPL), while OCTA and clinical data, respectively provide quantitative indicators of microvasculature in the macular area and independent validation of systemic endpoints. This information integration path from imaging to clinical endpoints indicates that integrating UWF-CFP with OCTA and clinical table data can synergistically enhance the risk assessment ability for systemic complications at different resolution levels. Overall, extending fundus imaging to systemic risk prediction has biological rationality, but prospective studies directly based on UWF-CFP are still relatively scarce. Future dedicated multicenter prospective studies are needed to confirm its independent predictive efficacy for hard endpoint events.

### 5.4 Technical challenges and generalization

Most current AI models for UWF-CFP are trained and validated on data from a single device and a specific population, which severely limits their generalizability. There are currently several technologies that can effectively address this shortcoming. (1) Domain Adaptation Technology: It reduces the feature differences between different devices and different populations to enable model migration and adaptation across scenarios. This is especially suitable for multi-model domain adaptation algorithms for DR classification (58). (2) Federated Learning: this approach supports multi-center training without sharing raw images. For instance, the DRFL framework trained a DR grading model across five clients and achieved 98.6% accuracy, confirming the feasibility of privacy-preserving collaboration (59). (3) Transfer Learning: leverages large-scale pre-trained models to improve generalization when UWF-CFP data are limited. An active learning framework with soft-label denoising reached 91.2% accuracy in cross-domain DR classification (60). However, these strategies have so far been validated mainly on traditional posterior-pole color fundus images, and systematic application to UWF-CFP remains lacking. Beyond algorithmic generalization, clinical translation also requires a complete data-processing pipeline. The overall process can be divided into three core steps: (1) Front-end input: complete UWF-CFP acquisition and standardized preprocessing, which includes cropping regions of interest (20), applying histogram equalization, and normalizing image resolution; (2) Feature extraction: through modality-specific encoders, hierarchical extraction of information related to diabetic retinopathy is achieved. EfficientNetB4, for instance, offers a good balance between training and validation accuracy (61); (3) Back-end prediction: after integrating the extracted features with complementary multimodal data, they are input into the classifier or large language model to generate DR grading and diagnostic outputs. Alwakid et al. demonstrated that applying CLAHE and ESRGAN for image enhancement raised DR grading accuracy from 80.87% to 98.7%, highlighting how front-end preprocessing can decisively influence downstream prediction (62). Clarifying this complete workflow is helpful for targeted solutions to problems such as image quality differences among different devices in DR screening, spatial misalignment between UWF-CFP and other modalities, and limitations in computational efficiency in large-scale DR screening scenarios.

The true potential of UWF-CFP is not presented in isolation, but is revealed within a broader diagnostic ecosystem through the integration of complementary data streams such as OCTA microstructure, metabolic indicators, and functional information. This integration can overcome and balance the inherent limitations of single-modalities such as field of view and resolution, structural information and functional data (63). By integrating UWF-CFP with OCTA and clinical table data, the accuracy of DR grading diagnosis can be enhanced, but the extent of this improvement varies significantly across different studies. Technological advancements in core architecture such as heterogeneous encoders, hierarchical fusion, cross-modal attention mechanisms, and self-supervised pre-training have collectively contributed to this performance improvement. Apart from the model architecture, rigorous data processing methods are equally crucial. Improper data set division can lead to data leakage, which is an easily overlooked issue. Patient-level data partitioning: all images from the same patient are specifically assigned to the training set or validation set. Combined with stratified sampling and a fixed random seed, this prevents performance inflation caused by random image-level segmentation and ensures that model evaluation reflects the true generalization ability (64, 65). Extending fundus images to systemic risk prediction has biological rationality, but clinical translation still faces multiple challenges such as insufficient validation and lack of standardization. Future research is needed to conduct prospective multicenter studies to verify the clinical efficacy of these fusion strategies and establish standardized multi-modal assessment frameworks.

## 6 Discussion

The premise of this review stems from the fundamental transformation currently taking place in the assessment system for DR. The traditional ETDRS 7-field imaging only covers approximately 35% of the retina, while the emergence of UWF-CFP has made it possible to visualize the peripheral retina. At the same time, the rapid development of deep learning and multimodal data fusion technologies has created new opportunities for automated analysis and overall risk stratification. However, these current research trajectories are largely still advancing independently, and there is an urgent need for integration. This discussion section will conduct a critical examination of this situation in order to outline a clear path for the clinical translation of UWF-CFP.

### 6.1 Challenges, limitations, and research gaps of UWF-CFP in DR management

Although UWF-CFP has significant advantages in DR assessment, it still faces several challenges in clinical applications. Firstly, in terms of imaging technology, the pseudo-color imaging used by cSLO systems such as Optos^®^ has relatively poor color fidelity for hard exudates, cotton-wool spots, and superficial hemorrhages. This may lead to systematic underestimation of lesion extent and affect the accurate judgment of non-proliferative DR severity (14, 17). Additionally, differences among devices from different manufacturers (e.g., Optos^®^ versus Zeiss Clarus^®^) in field of view, color mode, and image resolution pose challenges for data integration across multicenter studies and comparability in longitudinal patient follow-up (15). Common image artifacts in UWF-CFP, such as eyelid shadows, blurred eyelashes or motion artifacts, can significantly affect image interpretation. However, there is currently no consensus on how to handle or eliminate such images. Secondly, there is significant inter-reader variability in the interpretation of peripheral retinal lesions, especially when distinguishing IRMA from NVE. IRMA indicates severe NPDR, while NVE marks progression to PDR, a key distinction that determines different treatment pathways. Currently, there is no standardized reading protocol or validated reference atlas for peripheral lesion grading. Thirdly, there is a significant lack of standardized quantitative tools for evaluating the peripheral retina. Most existing AI software focuses on the posterior pole, as lesion features in this region are easier to segmented. Consequently, quantitative analysis of peripheral regions largely relies on manual grading or semi-quantitative scales, limiting the reproducibility and comparability of results across studies and centers. Fourthly, regarding the applicable population, although UWF-CFP has some penetrating ability in cases of mild media opacity, the imaging failure rate increases significantly with severe cataracts, corneal opacities, or vitreous hemorrhage. In such cases, traditional examination methods may still be required in marginal cases (21). Fifthly, in terms of clinical evidence, most studies linking UWF-CFP vascular parameters to diabetic kidney disease or cardiocerebrovascular complications are retrospective analyses based on traditional fundus photography. Large-scale prospective cohort validation remains lacking, and its independent predictive value as a non-invasive tool for systemic complications risk assessment requires further confirmation (54, 55). Sixthly, from a health economics perspective, the high acquisition cost of UWF-CFP equipment and the high examination expenses limit its accessibility in primary healthcare institutions and resource-limited areas, thereby restricting its widespread application in large-scale DR screening (42). In terms of cost-effectiveness, a Thai randomized controlled study reported that UWF is more cost-effective compared to drug mydriasis (hospital perspective ICER = −13.87, social perspective ICER = 76.46), but no formal cost-effectiveness assessment was conducted in this scenario (66). To date, there are no studies that incorporate the combined screening function of UWF-CFP for ocular and systemic complications into economic models, nor have they evaluated its cost-effectiveness in large-scale community screening scenarios. Seventhly, in terms of AI integration, existing deep learning models based on UWF-CFP are mostly trained on data from a single device and a single center, and their generalizability across different devices, ethnicities, and disease spectra has not been fully validated (40, 43). Although multi-model domain adaptation strategies have shown preliminary promise for cross-device transfer (44), their effectiveness in real-world multicenter settings requires further validation.

Apart from the model’s generalization ability, the transformation of the multimodal AI system based on UWF-CFP into routine practice is also hindered by unresolved research gaps. Currently, most of the evidence comes from retrospective studies, lacking long-term follow-up data on complication prediction, and the practical applicability of these models has not been fully verified. On the other hand, the integration of clinical workflow also faces certain challenges, including the lack of standardized image acquisition protocols, image artifacts, differences among readers, and the difficulty of integrating UWF-CFP into existing electronic health records. Therefore, before UWF-CFP is transformed from a research tool into standard clinical practice, these fundamental gaps in evidence and workflow integration must be addressed.

### 6.2 Tiered feasibility of UWF-CFP implementation across healthcare settings

The feasibility of implementing UWF-CFP varies significantly in different healthcare resource environments. In tertiary referral centers in middle- and high-income countries, it is recommended to fully deploy UWF-CFP as a solution for large-scale DR screening and stratification of systemic complication risks. For secondary hospitals or community health centers, UWF-CFP can be selectively deployed through government subsidies, cloud-based AI algorithms, and remote review networks of regional referral centers (67). In resource-limited primary healthcare settings, direct deployment of UWF-CFP is not feasible. However, by leveraging wide-angle retinal imaging on smartphones, image collection tasks can be undertaken by trained non-doctors, and support for offline operation can be provided by AI tools to expand the screening coverage (68). Finally, mobile screening vehicles equipped with UWF-CFP represent a promising model for serving remote and underserved populations, reducing barriers to healthcare access and improving patient compliance (69). By adopting a stratified and context-specific implementation strategy, combined with equipment sharing, AI automation, and telemedicine, UWF-CFP can be gradually expanded from tertiary centers to community medical institutions.

### 6.3 The imperative of explainable AI in UWF-CFP analysis

The “black box” nature of deep learning models remains the main obstacle for their clinical application. Explainable artificial intelligence (XAI) can enhance diagnostic accuracy and doctors’ trust in applications based on UWF-CFP (70). In XAI technology, gradient-weighted class activation mapping (Grad-CAM) and its variants are widely used to generate heat maps to highlight the image regions that have the greatest influence on the model’s predictions, enabling clinicians to confirm that the AI is focusing on clinically relevant areas such as microaneurysms or new blood vessels (71). Currently, there are no standardized XAI explanation evaluation indicators, and most studies rely on basic visual assessment rather than quantitative verification based on clinicians’ expertise (72). For UWF-CFP, there is an urgent need to conduct prospective studies to compare the effects of XAI-assisted reading with traditional reading, in order to quantify the additional clinical value of explainability, especially for the detection of peripheral lesions with significant prognostic significance (73).

### 6.4 Future perspectives: advancing UWF-CFP toward clinical translation and eye–system collaborative management

The clinical application of UWF-CFP is driving a fundamental transformation in the diagnosis and management of DR. This integrated framework is summarized in Figure 4, which illustrates the evolution from posterior pole–centered assessment to panretinal evaluation and ultimately to eye–system collaborative. UWF-CFP not only overcomes the blind spots inherent in traditional ETDRS seven-field photography and reveals the critical role of peripheral lesions in disease staging and progression, but also, through deep integration with ultra-widefield UWF SS-OCTA and AI technologies, achieves precise identification and quantitative analysis of key pathological changes such as non-perfusion areas and neovascularization under non-invasive conditions. At the level of AI integration, intelligent models based on UWF-CFP and deep learning have enabled automated DR screening and grading, as well as high-precision vessel segmentation of key pathological features. These parameters are not only closely associated with DR severity but also serve as an important bridge linking ocular pathology to systemic health, providing a non-invasive and quantifiable new pathway for risk warning of diabetic kidney disease and cardiocerebrovascular complications.

In this review, we tentatively propose the concept of “eye–system collaborative management” as a potential multidisciplinary care framework. This concept envisions UWF-CFP as a non-invasive window to assess the health of microvessels throughout the body, and it integrates retinal findings into the risk assessment and management of diabetes-related complications (such as diabetic nephropathy, cardiovascular diseases, and stroke). Its envisioned implementation would follow a three-step process: (1) Standardize and input the vascular parameters extracted from UWF-CFP (such as fractal dimension, peripheral hypoperfusion area) into the electronic health record; (2) Develop multimodal deep learning models that combine these parameters with clinical data to generate individualized risk scores for systemic complications; (3) Establish a structured nursing pathway, triggering early specialist referrals or intensified interventions based on the risk score, and providing support through regular multidisciplinary discussions among ophthalmologists, endocrinologists, nephrologists, and cardiologists. It must be emphasized that this framework remains conceptual at this stage. Its clinical feasibility and added value over existing risk-prediction models require prospective validation.

Future research should focus on several key directions. Firstly, a standardized image acquisition and lesion annotation protocol across different UWF-CFP devices should be established (74), and a multi-center prospective cohort covering multiple ethnic groups should be constructed to verify the predictive value of UWF-CFP-derived biomarkers for DR progression and systemic complications. Secondly, developing a multimodal deep learning model integrating UWF-CFP, ultra-wide-angle UWF SS-OCTA, and clinical data is crucial for improving diagnostic accuracy. Thirdly, enhancing the generalization ability of algorithms in different devices and ethnic/racial groups through domain adaptation and federated learning, while improving model interpretability, is necessary to promote clinical adoption. Fourthly, long-term prospective studies are needed to confirm the association between UWF-CFP parameters and hard outcome indicators such as end-stage renal disease, myocardial infarction, and stroke, thereby establishing its role in non-invasive global risk stratification. Finally, rigorous cost-effectiveness analysis and the development of portable ultra-wide-angle devices are crucial for converting these technologies into routine diabetes management (75), particularly in resource-limited settings, ultimately testing the feasibility of a potential paradigm shift toward what we have termed “eye–system collaborative management.”

### 6.5 Quantitative trend interpretation and its evidence boundaries

The quantitative trends extracted in this review must be interpreted within the limitations of the existing evidence. To date, most of the included studies adopt cross-sectional or retrospective designs. Even the existing prospective cohorts only tracked the progression of DR and did not follow up on the occurrence of systemic events such as end-stage renal disease or stroke. Therefore, the observed association between UWF-CFP parameters and systemic complications should be regarded as an hypothesis-generating nature rather than confirmatory evidence. Although the risk of DR deterioration related to PPL is between 1.7 and 4.7 times, the κ-value of UWF SS-OCTA consistency with FFA is concentrated in the high range of 0.869–0.916, and the OR values of systemic complications are all positive (1.47–11.36), these trends do not constitute formal combined estimates. Due to significant heterogeneity in imaging equipment, grading standards, and outcome definitions among the studies, meaningful meta-analyses cannot be conducted. The highest level of evidence currently available is only in the field of DR lesion detection (comparison of UWF-CFP combined with UWF SS-OCTA and FFA, κ = 0.916). However, predictive prospective data for new-onset stroke, end-stage renal disease, or myocardial infarction are almost completely lacking. Therefore, establishing a unified reporting standard and conducting multicenter prospective studies is a prerequisite for moving from qualitative synthesis to definitive quantitative conclusions.

## 7 Conclusion

The UWF-CFP has shifted the DR assessment from the posterior pole-centered model to a panretinal perspective, significantly improving the detection rate of peripheral lesions and enhancing the accuracy of disease grading. The current main challenges include imaging differences between devices, the lack of peripheral quantitative tools, significant inter-reader variability, and the insufficient generalization ability of AI models across devices and populations. The core advantage of UWF-CFP lies in its potential as a non-invasive observation window for overall microvascular health. Its vascular parameters have shown associations with diabetic nephropathy, cardiovascular diseases, and stroke risk. The proposed “eye–system collaborative management” concept in this paper envisions integrating retinal imaging markers with systemic clinical data to achieve individualized risk stratification. However, this framework is still at the conceptual stage and requires prospective validation. In the future, standardized collection protocols, multi-center prospective cohorts, domain adaptation and federated learning applications, as well as health economics evaluations, will jointly promote the realization of its clinical value.
