# Supplementary material for: Ultra-widefield color fundus photography in diabetic retinopathy: from panretinal assessment to multimodal integration
Source: Front Med (Lausanne). 2026 Jun 3;13:1845156. doi: 10.3389/fmed.2026.1845156 (PMC13271908; doi:10.3389/fmed.2026.1845156)
Supplement: Supplementary file 1 [file Data_Sheet_1.docx]

Supplementary Material

# Supplementary Figures


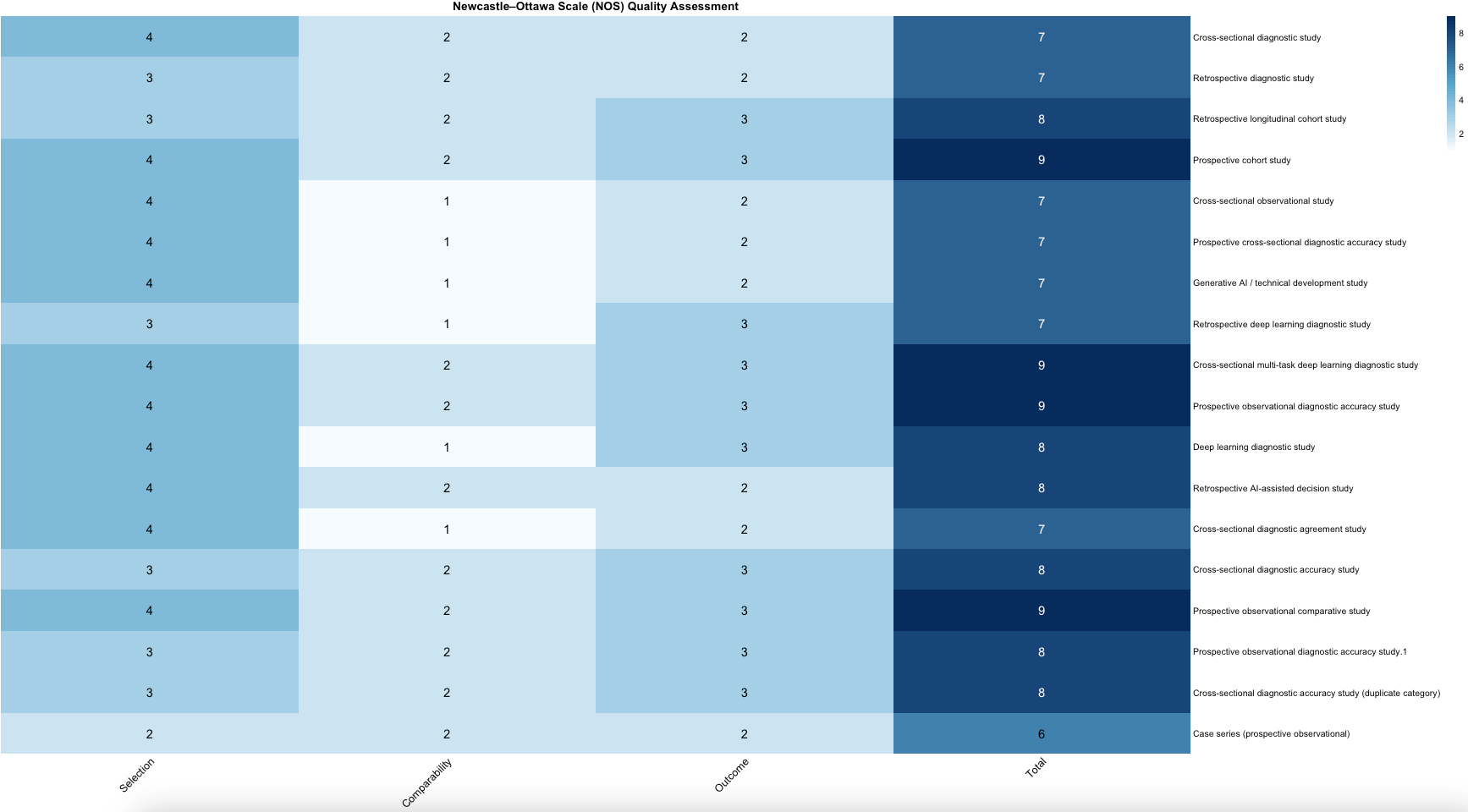


**Supplementary Figure 1.** **Newcastle‑Ottawa Scale (NOS) Quality Assessment of studies included in the review.** The heatmap presents the methodological quality scores of individual studies assessed using the Newcastle‑Ottawa Scale adapted for cohort, case‑control, and cross‑sectional diagnostic accuracy studies. Scores are shown across three domains: Selection (maximum 4 points), Comparability (maximum 2 points), and Outcome/Exposure (maximum 3 points), with total scores ranging from 0 to 9. Study designs are annotated on the right, including prospective cohort studies, retrospective longitudinal cohort studies, cross‑sectional diagnostic accuracy studies, retrospective AI‑assisted decision studies, and case series. Higher total scores (e.g., 8–9) indicate better methodological quality.
